# The metastatic spectrum in functional and non-functional NENs: mechanistic insights from multi-omics

**DOI:** 10.3389/fendo.2026.1782791

**Published:** 2026-08-27

**Authors:** Zhengjian Wang, Xuda Ji, Zhe Wang, Shunzhen Zheng, Fangfeng Liu, Jun Lu, Hong Chang

**Affiliations:** 1 Department of Hepatobiliary Surgery, Shandong Provincial Hospital Affiliated to Shandong First Medical University, Jinan, Shandong, China; 2 Shandong Provincial Hospital, Shandong University, Jinan, Shandong, China

**Keywords:** biomarker validation, functional NETs, metastasis, multi-omics integration, neuroendocrine neoplasms, non-functional NETs, pancreatic neuroendocrine tumors, recurrence

## Abstract

Neuroendocrine neoplasms (NENs) are biologically heterogeneous tumors in which differentiation/grade and hormonal functionality are intersecting but non-equivalent axes. This review focuses on functional and non-functional well-differentiated neuroendocrine tumors (NETs), principally gastroenteropancreatic and pancreatic NETs, and critically evaluates how site, lineage, stage, tumor burden, genomic and epigenetic alterations, immune-stromal remodeling, metabolic adaptation, microbiome-associated signals, and treatment pressure converge on metastasis and recurrence. Apparent outcome differences by functionality are inconsistent after clinicopathological adjustment: non-functional presentation is often enriched for delayed diagnosis and adverse features, whereas functional subtypes range from typically indolent insulinomas to clinically aggressive hormone-producing tumors. We reconcile these observations through a layered model in which lineage-defining alterations and chromatin/telomere programs establish cellular state; signaling and metabolic plasticity enable stress adaptation; and hypoxia, angiogenesis, immune cells, fibroblasts, extracellular matrix, and therapy create selective niches for dissemination and relapse. We also define computational strategies for heterogeneous multi-omics integration and a staged biomarker-validation pathway. Evidence remains dominated by pancreatic NETs, and causal support is weakest for microbiome-functionality relationships and several proposed cross-omic links. A spectrum-based framework is therefore most useful when it generates testable, site- and grade-specific hypotheses rather than treating functionality as an isolated prognostic variable.

## 1 Introduction

Neuroendocrine neoplasms (NENs) comprise site-specific diseases that share neuroendocrine differentiation but differ in lineage, genomic architecture, natural history, and treatment sensitivity. The 2022 WHO framework separates well-differentiated neuroendocrine tumors (NETs) from poorly differentiated neuroendocrine carcinomas (NECs) and grades NETs by proliferative activity (1). Within NETs, functionality is defined by a clinically relevant hormone-excess syndrome supported by biochemical evidence; hormone immunoreactivity or peptide secretion without a syndrome is insufficient (2–4). This review examines metastasis and recurrence in functional and non-functional NETs, with emphasis on gastroenteropancreatic and pancreatic disease. Functional syndromes can trigger diagnosis at low tumor burden, whereas non-functional lesions may remain occult until mass effect or metastatic spread (2, 5, 6). The central question is therefore not whether one label is uniformly more aggressive, but whether functionality retains biological information after primary site, lineage, grade, stage, tumor burden, hereditary background, and ascertainment have been considered.

To move beyond descriptive comparison, we distinguish three levels of evidence: direct comparisons in site-, grade-, and stage-matched NET cohorts; NEN-specific multi-omics or experimental studies that define a progression mechanism without directly comparing functionality; and indirect evidence extrapolated from other neuroendocrine or non-neuroendocrine cancers. Molecular associations are treated as causal only when supported by temporal sampling, perturbation, lineage tracing, mediation analysis, or independent replication. This hierarchy is important because many studies compare unmatched clinical groups, analyze a single omics layer, or infer mechanism from cross-sectional bulk tissue (7–17). Throughout the review, we identify where findings converge, where results conflict, and which experiments would resolve the uncertainty.

### 1.1 Scope and terminology of this review

The primary comparison is between functional and non-functional well-differentiated NETs. Functional NETs are defined by a clinically evident hormone-excess syndrome with biochemical support; non-functional NETs lack such a syndrome despite possible hormone expression or subclinical secretion (1, 2, 5). NECs are retained as a separate high-grade comparator only when a study specifically addresses NEC biology, such as TP53/RB1 co-inactivation. Germline predisposition is also separated from somatic evolution because inherited MEN1, VHL, NF1, TSC, CDKN1B, RET, SDHx, or MAX alterations can influence age, multiplicity, surveillance, and endocrine phenotype without defining a universal functional state (18–20). The evidence base is explicitly pancreatic NET-dominant. Findings from small-intestinal, rectal, bronchopulmonary, thymic, pituitary, or pheochromocytoma/paraganglioma cohorts are therefore treated as site-specific evidence rather than pooled as biologically equivalent NEN observations.

## 2 Direct comparison of core concepts and clinical features

### 2.1 Clinical heterogeneity, diagnosis, and treatment of functional and non-functional NETs

Functional and non-functional NETs differ most reproducibly in clinical presentation and diagnostic timing. Functional syndromes such as hypoglycemia, acid hypersecretion, secretory diarrhea, or carcinoid syndrome prompt biochemical localization and often lead to investigation at lower tumor burden (2–4). However, the functional category is internally heterogeneous: insulinomas usually dominate surgical series and often have favorable outcomes, whereas gastrinomas, glucagonomas, VIPomas, and serotonin-producing tumors can present with nodal or distant disease (2, 3, 14, 21, 22). Non-functional pancreatic NETs are more common in most contemporary series, but estimates vary with referral patterns, hereditary surveillance, tumor-size thresholds, and the operational definition of a hormone syndrome (4, 21). These differences in ascertainment must be separated from intrinsic metastatic biology.

Diagnostic evaluation should verify the hormone syndrome, establish differentiation and grade, define anatomical extent, and characterize somatostatin-receptor expression when clinically relevant (2–4). For non-functional pancreatic NETs, cross-sectional imaging, receptor imaging, and selected radiomic models can refine localization and risk assessment, while systemic inflammatory indices provide associative rather than treatment-predictive information (6, 23–27). Management ranges from surveillance of carefully selected small non-functioning pancreatic NETs to surgery, somatostatin analogs, targeted therapy, peptide receptor radionuclide therapy (PRRT), chemotherapy, or liver-directed treatment according to site, grade, growth rate, receptor status, burden, symptoms, and resectability (4, 28–30). None of these diagnostic or therapeutic pathways establishes functionality as a stand-alone predictor of response.

### 2.2 Clinical patterns of metastasis and recurrence

Most unadjusted pancreatic NET series report that non-functional tumors are larger and more often have nodal involvement, vascular or perineural invasion, higher grade, or distant metastasis at diagnosis (11–14, 21, 23). Adjustment does not yield a uniform answer. One surgical cohort retained non-functional status as an independent recurrence factor (11), whereas other recurrence models are dominated by grade, nodal status, size, vascular invasion, and margin or stage variables (12–15, 23). The apparent conflict is clinically plausible: studies use different functional definitions, disproportionately compare insulinomas with all non-functional tumors, include different size and stage distributions, and apply different surveillance and treatment pathways. Residual confounding, lead-time bias, referral enrichment, and limited event numbers can therefore change the estimated effect of functionality.

Recurrence after resection most often involves the liver and lymph nodes, but the literature does not demonstrate a functionality-specific route of dissemination (12, 15). The favorable signal in many functional cohorts is largely insulinoma-weighted; it should not be generalized to all hormone-producing tumors. For example, a recent small molecularly characterized glucagonoma series documented frequent vascular, perineural, nodal, and distant spread despite well-differentiated histology (22). Conversely, selected small non-functional tumors remain indolent. Functionality may therefore encode lineage or detection information in a specific subtype, but its prognostic contribution is conditional rather than constant across NETs (2, 3, 11–15, 21).

The key clinical knowledge gap is a direct, adequately powered comparison that uses a common definition of functionality and prospectively records primary site, specific hormone syndrome, grade, stage, size, hereditary status, treatment, surveillance intensity, and time-dependent changes in secretion. Such a study should report both unadjusted and prespecified adjusted effects, test interactions between functionality and site or grade, and distinguish recurrence prediction from causal inference. Until then, functional status is best retained as one annotated variable within a clinicopathological model rather than removed or elevated to a dominant axis.

## 3 Molecular programs associated with malignant progression

### 3.1 Key mutational profiles

**Figure 1:**
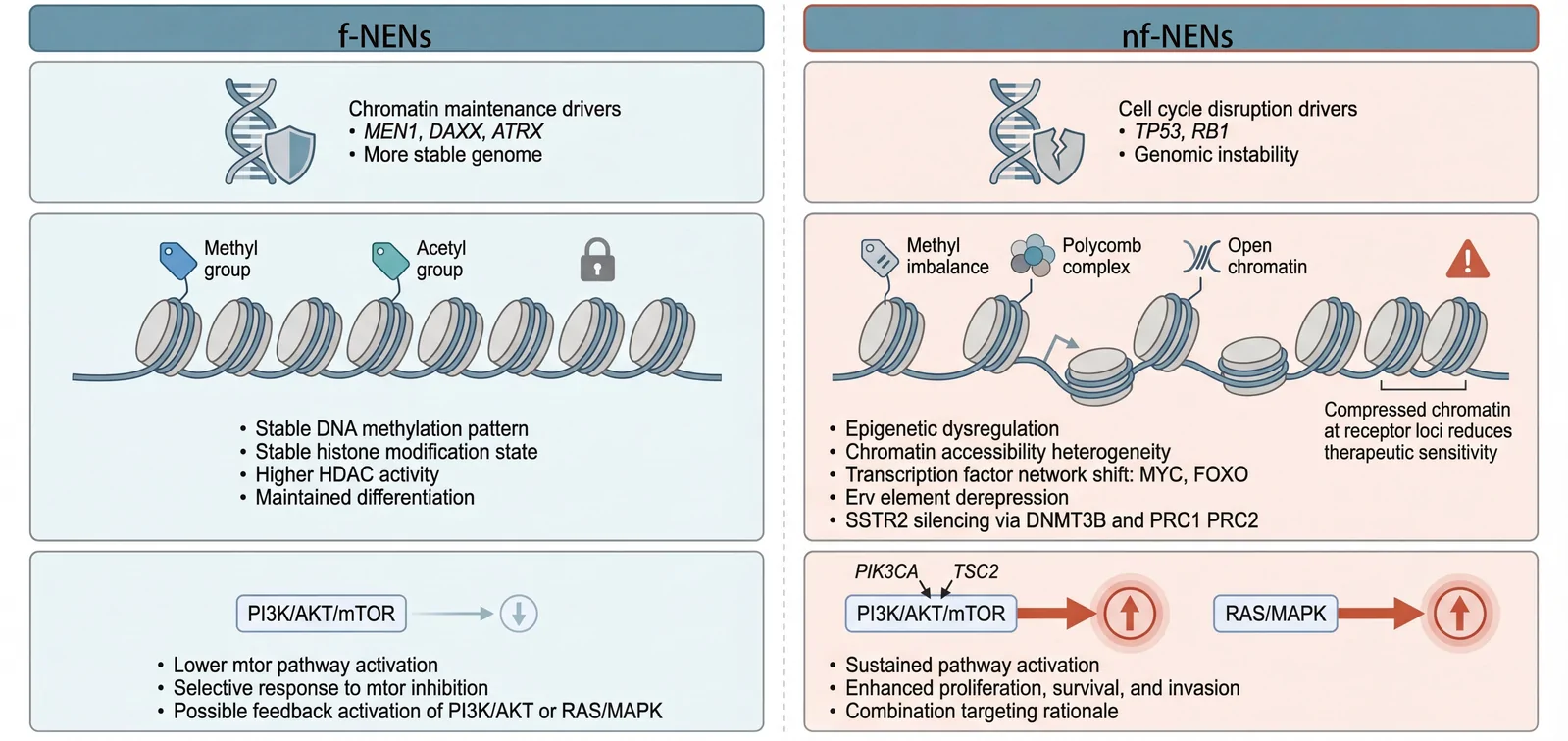
Genomic and epigenetic features across functional and non-functional NETs. MEN1/DAXX/ATRX alterations are common in well-differentiated PanNETs across functional categories, whereas TP53/RB1 alterations are mainly associated with poorly differentiated NECs or high-grade transformation. This framework emphasizes overlap between f-NETs and nf-NETs while keeping functional status distinct from differentiation and grade.

As summarized in **Figure 1**, MEN1, DAXX, ATRX, and mTOR-pathway alterations are recurrent in well-differentiated pancreatic NETs across functional categories and connect chromatin regulation, telomere maintenance, and growth signaling (10, 31–33). An important apparent contradiction concerns DAXX/ATRX. Some mutation-defined sequencing cohorts reported comparatively favorable survival, whereas loss of DAXX/ATRX protein and alternative lengthening of telomeres (ALT) in resected cohorts have been associated with chromosomal instability, metastasis, or shorter recurrence-free survival (10, 31–33). These results are not necessarily incompatible: genotype, protein loss, and ALT are related but non-identical measurements, and the cohorts differ in stage, treatment, endpoint, and survivorship. DAXX/ATRX should therefore be modeled as part of a lineage-telomere state rather than interpreted as a simple marker of functionality or prognosis.

TP53 and RB1 co-inactivation instead characterizes many poorly differentiated NECs and selected high-grade transformations, not non-functionality itself (34–40). This distinction has practical consequences because a non-functional well-differentiated NET and a poorly differentiated NEC require different prognostic and therapeutic frameworks. Germline MEN1 and other inherited syndromes influence the field in which tumors arise, whereas somatic copy-number evolution, telomere maintenance, and treatment selection shape subsequent progression (18–20). Integrating germline, somatic, lineage, and pathology data is therefore more informative than assigning individual alterations to a functional/non-functional binary.

### 3.2 Epigenetic regulation

Mechanistic integration begins where genomic alterations modify chromatin. MEN1 loss perturbs transcriptional and histone-regulatory complexes, while DAXX/ATRX loss can couple altered heterochromatin, DNA methylation, ALT, and chromosomal instability (32, 41–45). These processes can stabilize lineage states or increase evolutionary capacity, thereby changing the probability that a clone acquires invasive, stress-tolerant, or therapy-resistant phenotypes. The relevant unit is consequently a genome-epigenome state, not an isolated mutation list.

Methylation and chromatin studies identify pancreatic NET subgroups associated with lineage, copy-number pattern, and clinical behavior, but direct functional versus non-functional comparisons remain sparse (41–45). Bulk-tissue profiles also mix tumor cells, fibroblasts, endothelial cells, and immune populations, so an apparent epigenetic subtype may partly reflect tissue composition. Moreover, retrospective studies differ in platform, tumor purity, primary versus metastatic sampling, and outcome definition. These limitations help explain why individual methylation or chromatin markers have not yet become reproducible functionality-specific biomarkers.

Single-cell and spatial epigenomic or transcriptomic approaches can separate malignant lineage programs from microenvironmental composition and place chromatin states at invasive fronts (17, 46–48). Their advantages are offset by dissociation bias, loss of fragile populations, limited capture efficiency, spatial spot mixing, batch effects, and small numbers of clinically annotated tumors. Matched primary-metastasis and longitudinal studies, coupled to perturbation in lineage-appropriate models, are required before MYC-, FOXO-, or other accessibility programs can be assigned a causal role in NET dissemination.

### 3.3 Activity of key signaling pathways

PI3K/AKT/mTOR, RAS/MAPK, Wnt/β-catenin, and receptor-tyrosine-kinase networks connect growth signals to nutrient sensing, hypoxia adaptation, autophagy, lipid metabolism, and ferroptosis (49–56). mTOR inhibition can release feedback activation of AKT or MAPK, while hypoxia-driven metabolic pathways can reinforce mTOR signaling and survival. This bidirectional coupling provides a mechanistic bridge from genomic and epigenetic states to metabolic plasticity and treatment escape.

The literature therefore supports an adaptive signaling network rather than a sequence of independent pathways. Clinical activity of mTOR-directed therapy across functional categories is consistent with shared pathway dependence, but functionality has not been validated as a predictive biomarker (51–54, 57–59). A stronger test would combine phosphoproteomics, metabolic flux, and serial tumor or liquid-biopsy sampling before and during treatment to determine whether feedback modules, rather than baseline pathway abundance alone, predict resistance.

## 4 The shaping role of the tumor immune microenvironment

### 4.1 Immune cell infiltration patterns

**Figure 2:**
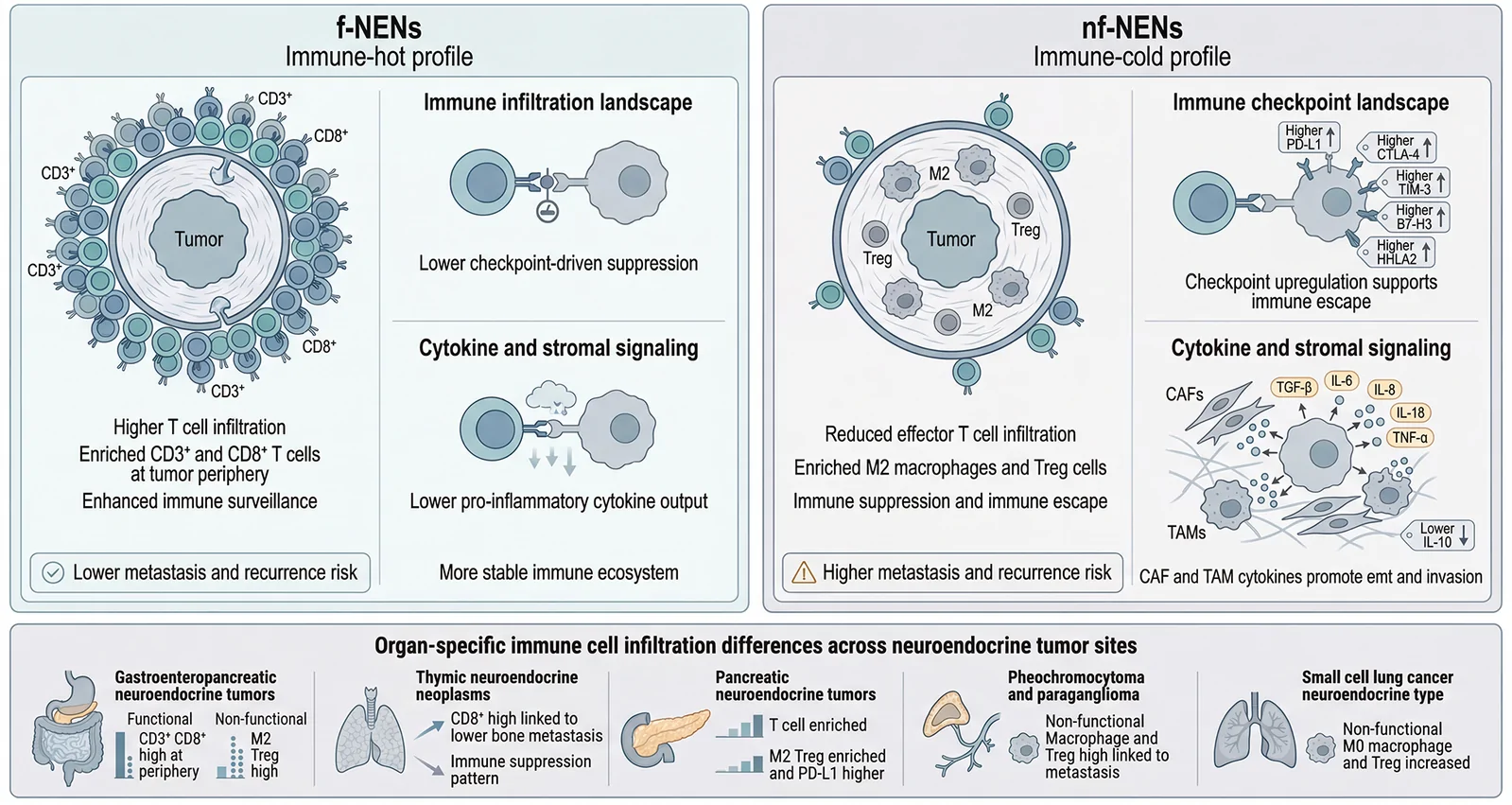
Overlapping immune microenvironment profiles in f-NETs and nf-NETs. Immune states form a spectrum shaped by grade, site, differentiation, tumor burden, stromal context, and functional status, rather than a binary immune-hot versus immune-cold division. T-cell infiltration, tertiary lymphoid structures, macrophage enrichment, checkpoint expression, and cytokine profiles may vary across NEN subgroups.

Immune organization varies markedly across NEN sites. The conceptual immune landscape is summarized in **Figure 2**. In resected non-functional pancreatic NETs, tertiary lymphoid structures and T-cell infiltration have been associated with favorable outcome (16). Duodenal NETs, thymic NENs, pheochromocytoma/paraganglioma, and pituitary NETs show different lymphoid and myeloid patterns (60–63). These observations broaden the evidence beyond the pancreas, but their disagreement is informative: primary site, lineage, grade, and local tissue ecology appear to dominate any common immune signature of functionality.

Some advanced or high-grade NENs contain M2-like macrophages, regulatory T cells, or other suppressive myeloid populations, whereas selected well-differentiated tumors remain sparsely infiltrated (17, 63–65). Bulk deconvolution cannot reliably distinguish low cell density from altered cell state, and single-cell studies can overrepresent populations that survive tissue dissociation. Quantitative pathology, spatial profiling, and standardized sampling of primary and metastatic sites are therefore needed to determine whether immune states mediate recurrence or simply track grade and burden.

### 4.2 Immune checkpoint molecule expression

PD-L1 positivity is detectable in subsets of pancreatic and pituitary NETs and is generally more frequent in poorly differentiated or high-grade disease, but prevalence varies with antibody, scoring compartment, cutoff, specimen type, and prior therapy (65–68). These assay and case-mix differences preclude a stable functional/non-functional checkpoint pattern.

CTLA-4, TIM-3, B7-H3, and HHLA2 extend the candidate checkpoint landscape (69, 70), yet expression alone has not predicted benefit from immune-checkpoint blockade. The heterogeneous activity observed with atezolizumab plus bevacizumab further argues that grade, immune context, angiogenesis, and tumor site must be integrated (71). Prospective studies should lock assay methods and test a composite immune-stromal biomarker against a treatment interaction, rather than correlate multiple checkpoints with outcome after the fact.

### 4.3 Cytokine profiles and functional effects

Circulating and tissue cytokines provide another possible bridge between tumor state and systemic phenotype. IL-6, IL-8, IL-10, IL-17, IL-18, TNF-related signaling, and CSF-1-dependent macrophage programs have been associated with grade, disease status, or outcome in selected NEN cohorts (17, 72–75). However, blood concentrations may reflect hormone syndromes, liver metastases, infection, metabolic disease, or treatment, and cross-sectional data cannot identify the producing cell or direction of effect.

A biologically integrated model is more plausible than assigning individual cytokines to functionality: hypoxia, necrosis, or therapy can activate tumor cells, cancer-associated fibroblasts, and macrophages; TGF-β, IL-6/STAT3, and related signals can then reinforce extracellular-matrix deposition, immune exclusion, epithelial-mesenchymal-like programs, and stress tolerance (17, 74–76). Tumor-derived signals in turn maintain the stromal state. Demonstrating this feedback requires paired spatial measurements and perturbation of the inferred ligand-receptor axis, not serum association alone.

## 5 Metabolic-microbial mechanisms potentially associated with differential invasiveness of f-NETs and nf-NETs

### 5.1 Metabolic reprogramming

**Figure 3:**
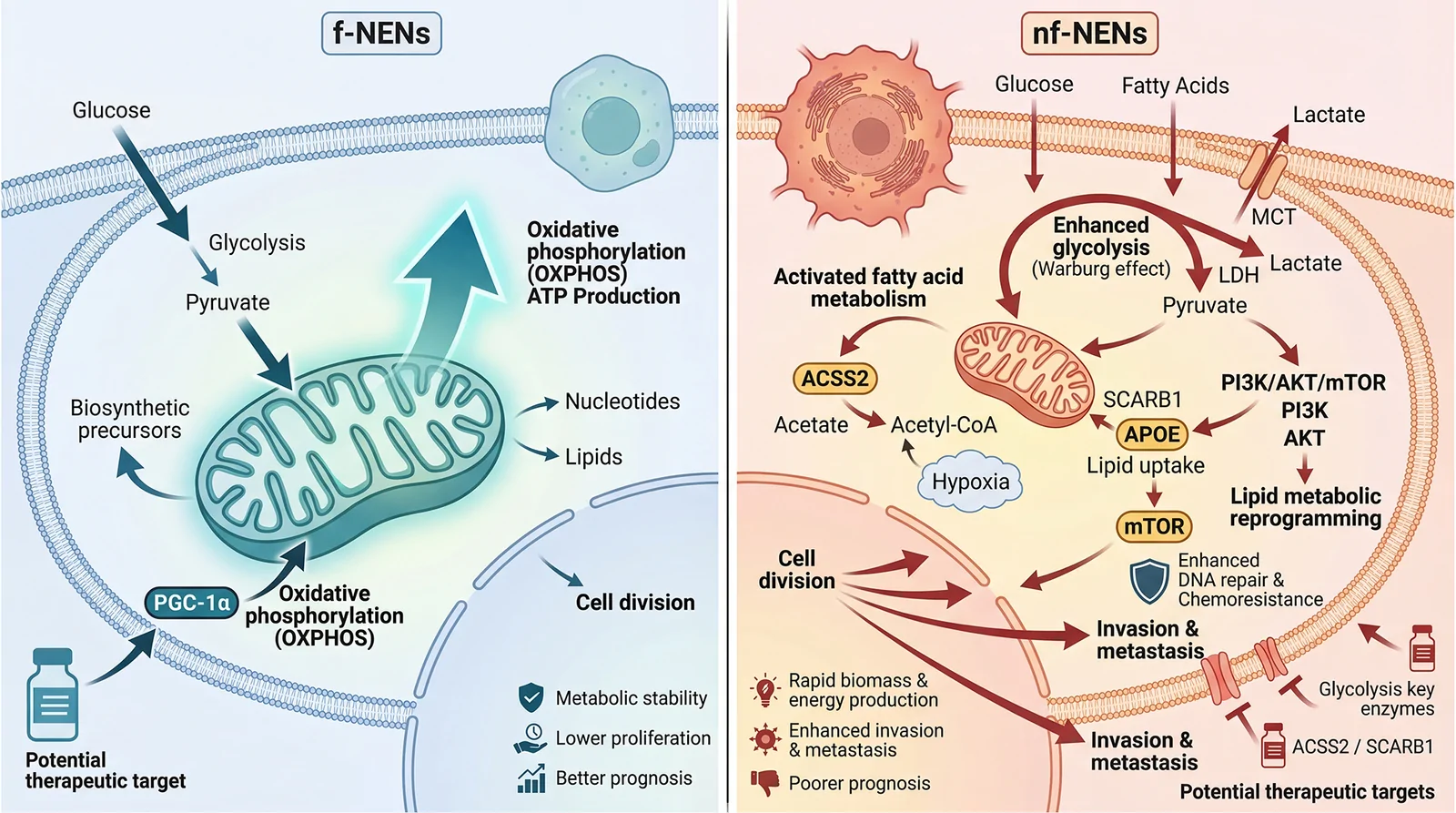
Potential metabolic reprogramming patterns in f-NETs and nf-NETs. Glycolysis, oxidative phosphorylation, lipid metabolism, acetylation-related regulation, and hypoxia-driven pathways are context-dependent programs. These features may be enriched in selected high-risk or non-functional cohorts, but they are unlikely to be determined by functional status alone.

Metabolic reprogramming in NENs includes glycolysis, mitochondrial oxidative phosphorylation, lipid uptake and synthesis, amino-acid use, redox control, and metabolite-sensitive epigenetic regulation (55, 77–83). Reported patterns differ among well-differentiated pancreatic NETs, pulmonary NEC models, and pituitary NETs, reflecting lineage as well as grade. Metabolomic conclusions are also sensitive to ischemia time, fasting and medication, sample matrix, platform coverage, normalization, and tumor-cell content. These sources of variation limit causal comparison by functionality. **Figure 3** summarizes these context-dependent metabolic programs.

Pancreatic NET models provide specific mechanistic examples. Hypoxia-induced ACSS2 and tumor-derived APOE can promote lipid remodeling or temozolomide resistance, whereas FASN inhibition and MEN1-mTOR-SCD1 signaling implicate ferroptosis-related vulnerabilities (55, 56, 81, 82). These studies establish targetable biology in selected models but do not show that the programs arise because a tumor is non-functional. Integration with genomic subtype, phosphoproteomics, spatial hypoxia, and treatment response, as demonstrated in recent proteogenomic work, is a more credible route to patient stratification (84).

### 5.2 Gut microbiota composition

Microbiome evidence is the least mature layer of the proposed framework. Cross-sectional studies in midgut, rectal, pituitary, and gastroenteropancreatic NENs report altered taxa or metabolites (85–90), but cohorts are small and differ in diet, geography, proton-pump inhibitors, antibiotics, somatostatin analogs, bowel surgery, hormone syndromes, and metastatic burden. A microbiome signature may therefore be a consequence of disease or treatment rather than a driver of metastasis.

The observation of microbiome-metabolome changes in both functional and non-functional pituitary NETs argues against a universal functionality-specific community (89, 90). Mendelian-randomization and association studies can prioritize hypotheses but cannot substitute for direct metabolite measurement and intervention (91). Likewise, altered circulating organic acids in NET patients do not identify a microbial source without paired metagenomic and flux data (92). Longitudinal sampling before and after treatment is essential to establish directionality.

### 5.3 Interactions between microbial metabolites and signaling pathways

A plausible mechanistic chain is that short-chain fatty acids, bile acids, or tryptophan metabolites alter epithelial-barrier function and systemic immunity, which then modulate NF-κB-, mTOR-, or cytokine-related signaling in tumor and stromal cells (56, 90, 92–94). Each link is biologically supported in broader host-microbe research, but the complete chain has not been demonstrated in a NEN model of metastasis.

**Figure 4:**
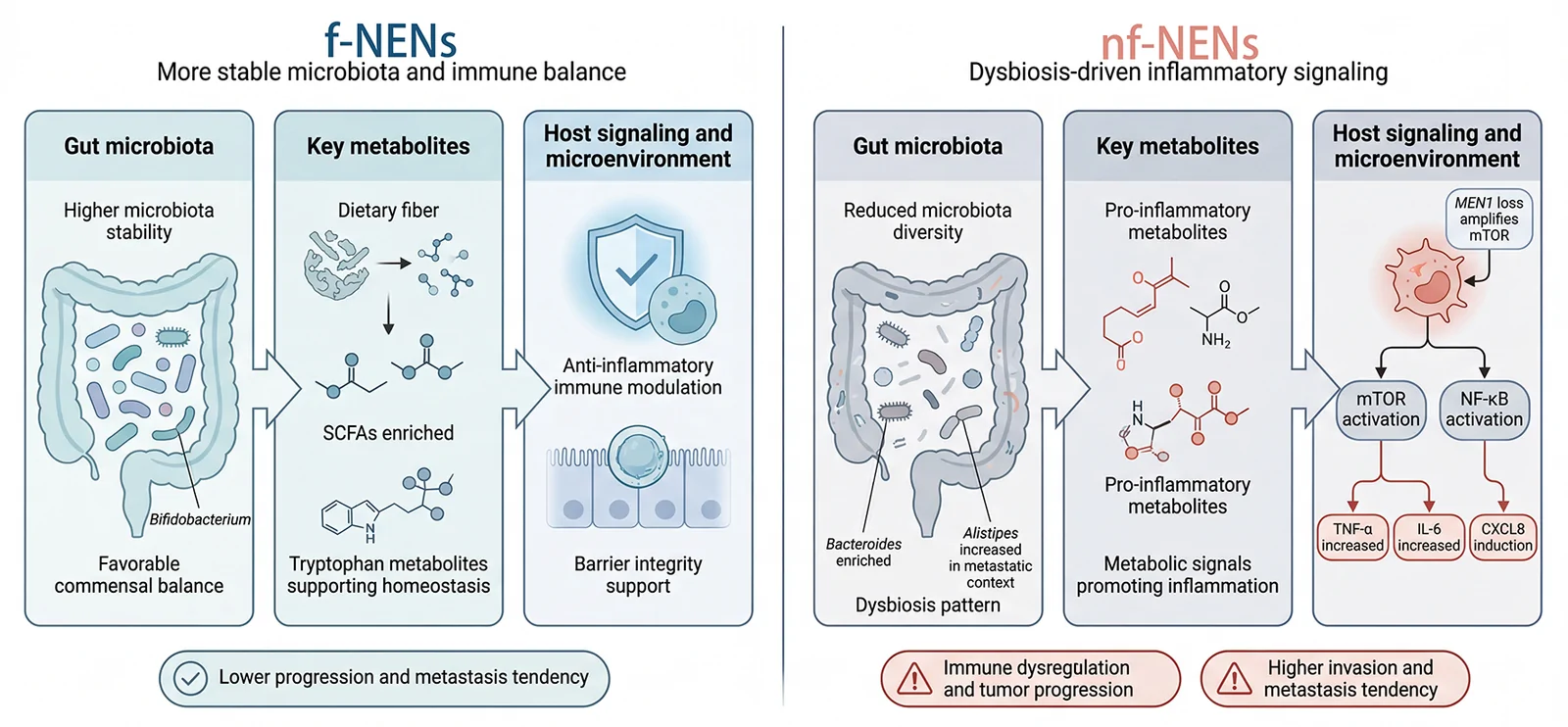
Gut microbiota-metabolite-host signaling axis in NENs. Microbiota, short-chain fatty acids, tryptophan-derived metabolites, inflammation, mTOR, NF-κB, and immune modulation form a hypothesis-generating network. Functional status-specific causality requires validation in longitudinal and mechanistic studies.

A minimal causal test should show temporal exposure, a defined microbial gene or metabolite, engagement of a host receptor or pathway in the relevant tissue, and reversal by microbial, dietary, metabolite, or receptor perturbation. Functional and non-functional NETs would then need comparison within the same site, grade, stage, medications, and diet. Until those conditions are met, **Figure 4** should be read as a hypothesis-generating host-microbe-signaling model rather than evidence that dysbiosis drives a particular functional phenotype.

## 6 Other key modifiers of progression

### 6.1 Angiogenic capacity

**Figure 5:**
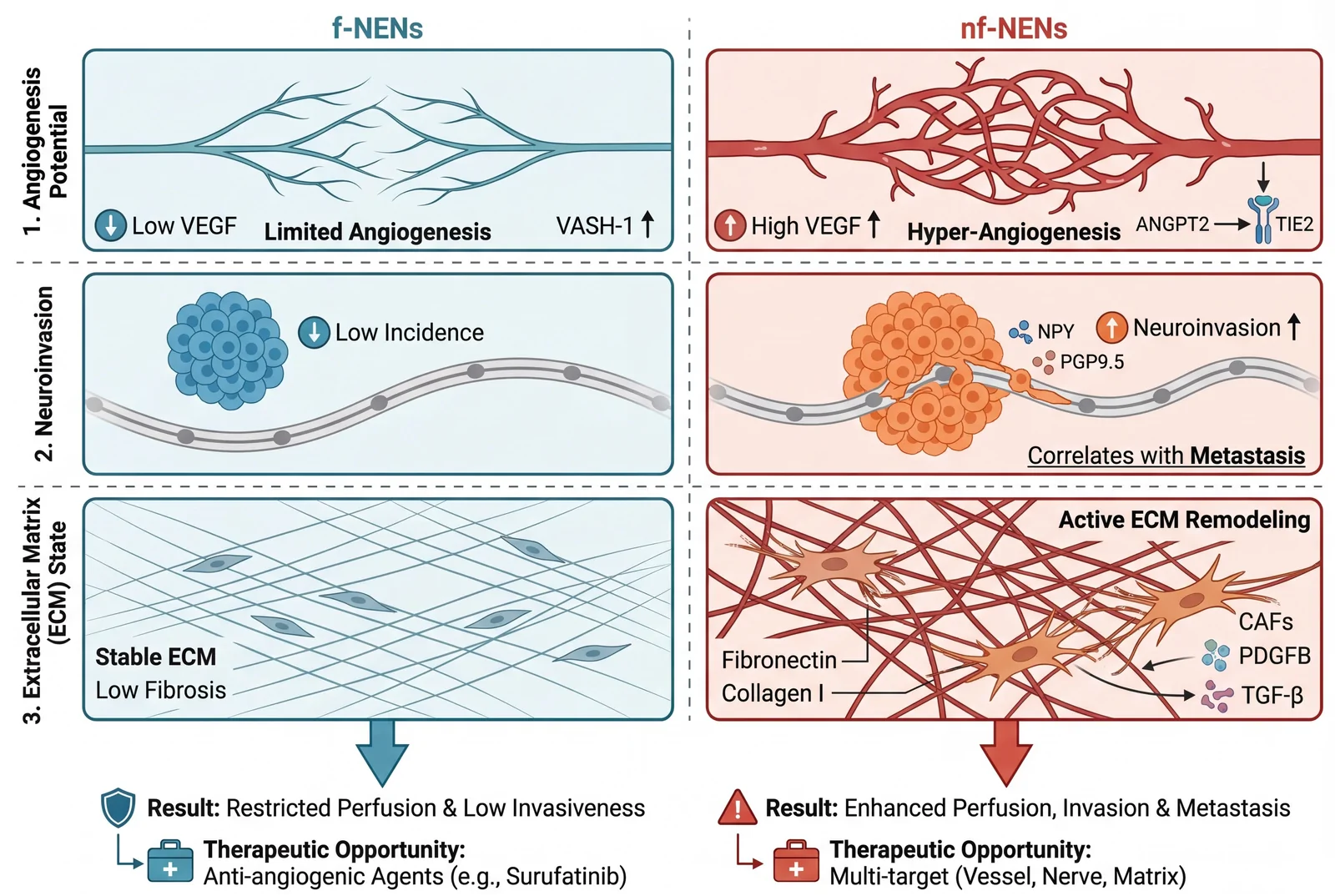
Angiogenesis, neuroinvasion, and extracellular matrix remodeling in NEN progression. VEGF-related angiogenesis, nerve-tumor interactions, CAF activation, PDGFB/TGF-β signaling, and ECM remodeling are progression-associated processes influenced by tumor site, grade, stage, and TME composition. Functional status may modify clinical presentation but is unlikely to act as the sole driver of these processes. Therapeutic implications are schematic and should not be interpreted as functionality-specific treatment recommendations.

As summarized in **Figure 5**, well-differentiated pancreatic NETs are often hypervascular, yet vascular density is not a linear surrogate for metastatic competence. Progressive regions can be hypoxic and poorly perfused despite VEGF-related signaling, and abnormal vessels may facilitate invasion while failing to normalize oxygen delivery. Spatial pancreatic NET data and experimental studies implicate VEGF-related pathways, RABL6A, NRP2, and ANGPT2/TIE2 signaling in this heterogeneous angiogenic state (17, 95–100).

This apparent vascular paradox links signaling, metabolism, and stroma: hypoxia selects stress-tolerant clones, induces metabolic remodeling, and shapes fibroblast and immune behavior, while angiogenic signaling creates dysfunctional routes for dissemination. Functional status has not been validated as a predictor of anti-angiogenic benefit. Treatment selection should instead follow clinical indication, disease tempo, prior therapy, and the molecular-vascular context (100, 101).

### 6.2 The role of perineural invasion

Perineural invasion is an adverse pathological feature in pancreatic NETs and is associated with nodal, vascular, or organ invasion and higher grade (102, 103). The available evidence is retrospective, depends on pathological sampling, and rarely includes a prespecified functionality analysis. Perineural invasion should therefore be modeled as an anatomical and microenvironmental mediator of recurrence, not as evidence that absent hormone secretion causes neural invasion.

Mechanistic claims about nerve-derived trophic signals in NETs remain less secure than the pathological association. Parts of the cancer-neuroscience literature are extrapolated from other tumor types (104, 105), while correlations involving CD56 or other neural markers do not establish active nerve-tumor signaling (106, 107). Nerve-resolved spatial profiling, electrophysiological or denervation perturbation, and matched functional subtypes are needed to distinguish neural route, lineage marker, and causal niche effects.

### 6.3 Extracellular matrix remodeling

Extracellular-matrix (ECM) remodeling connects fibroblast state, vessel function, immune exclusion, and invasion. Pancreatic NET studies implicate CAF-rich regions, collagen and fibronectin organization, PDGFB-related signaling, and TGF-β-associated programs, with some molecular differences in serotonin-producing tumors (17, 76, 108).

The evidence requires careful calibration. Spatial pancreatic NET data directly associate stromal programs with invasive and metastatic phenotypes (17), whereas experimental evidence that platelet-derived PDGFB controls CAF recruitment and matrix deposition was generated in a broader cancer model (76). The latter supports a candidate mechanism but does not demonstrate that PDGFB specifically drives non-functional NET metastasis. This distinction between NEN-specific observation and cross-cancer mechanism prevents overstatement.

ECM architecture may act as a mediator rather than a static correlate: hypoxia and cytokines activate fibroblasts; remodeled matrix changes perfusion, immune access, and cell migration; and the resulting stress feeds back to tumor signaling. Spatial multi-omics with matrix imaging and clone-resolved outcome data can test whether this loop adds prognostic information beyond grade, vascular invasion, and tumor burden (17, 109, 110).

### 6.4 Treatment response and drug resistance

Functionality primarily modifies symptom control and peri-treatment risk, whereas antitumor response is more consistently associated with differentiation, grade, somatostatin-receptor expression, burden, growth rate, molecular state, and prior therapy (71, 101, 111, 112). The phase 3 CABINET trial demonstrated cabozantinib activity in previously treated progressive pancreatic and extrapancreatic NET cohorts, illustrating a disease-context rather than functionality-based treatment question (101). Checkpoint, anti-angiogenic, and targeted combinations should likewise be evaluated with interaction biomarkers rather than *post hoc* functional subgroup labels.

Functional NETs may require simultaneous control of hormone excess and tumor growth. Somatostatin analogs, PRRT, targeted agents, chemotherapy, and liver-directed approaches are selected according to syndrome, receptor biology, grade, site, burden, and tempo (113–116). NETTER-2 supports first-line [177Lu]Lu-DOTATATE plus long-acting octreotide in selected advanced grade 2–3 well-differentiated gastroenteropancreatic NETs (115), but it does not establish functional status as the predictive variable.

Multi-omics, quantitative imaging, and organoid studies have generated candidate response mechanisms involving HRAS, filamin A, MYC, receptor imaging, and metabolic state (84, 117–121). Their translational value remains provisional because model establishment can select subclones, many studies lack an independent test cohort, and few signatures demonstrate treatment interaction or improvement over standard clinicopathological predictors.

## 7 Integrated analysis of metastasis and recurrence from a multi-omics perspective

### 7.1 A unified mechanistic model of metastatic progression and recurrence

A unified model begins with site-specific lineage and inherited or somatic drivers that establish chromatin, telomere, and transcriptional states. Under growth, hypoxic, and treatment stress, mTOR/MAPK feedback and metabolic plasticity permit survival. These tumor-cell states alter the secretome and recruit endothelial cells, fibroblasts, and myeloid populations; cytokines, abnormal vessels, nerves, and remodeled ECM then create spatially restricted routes for invasion. Disseminated clones encounter organ-specific niches and further selection by therapy. Functionality can change symptom detection and systemic physiology and may itself evolve, but it is not assumed to sit upstream of every metastatic mechanism (10, 17, 31–33, 41–55, 71, 76–84, 95–121).

Five reciprocal loops organize this model: genome-epigenome evolution, hypoxia-metabolic adaptation, immune-stromal remodeling, microbiome-host signaling, and therapy-driven clonal selection. Evidence is strongest for the first three loops in pancreatic NETs, intermediate for therapy-response mechanisms, and weakest for a causal microbiome-functionality link. **Table 1** converts the framework into measurable nodes, evidence levels, and validation experiments.

**Table 1:** Unified, testable multi-omics model of NEN metastasis and recurrence.

Mechanistic node	Cross-layer coupling	Current NEN evidence and causal strength	Testable readout/next validation step
Lineage, genome, and epigenome	MEN1/DAXX/ATRX and mTOR-pathway alterations shape chromatin, telomere maintenance, copy-number evolution, and lineage transcriptional states.	Recurrent and mechanistically coherent in PanNETs, but functional-status specificity is weak; genotype, protein loss, and ALT are not interchangeable (10, 31–33, 41–45, 84).	Joint mutation-IHC-ALT profiling in site-, grade-, and stage-matched cohorts; serial primary-metastasis sampling.
Signaling and metabolism	mTOR/MAPK feedback, hypoxia, lipid use, redox control, and ferroptosis couple proliferative signaling to stress adaptation and drug resistance.	Supported by PanNET models and selected patient cohorts, but direct functional versus non-functional comparisons are scarce (49–56, 77–84).	Phosphoproteomics plus isotope tracing and perturbation; test whether the integrated state predicts recurrence beyond grade and burden.
Immune-stromal-ECM niche	CAF/TAM cytokines, TGF-β/STAT3 signaling, checkpoint expression, collagen organization, and immune exclusion create reciprocal tumor-niche feedback.	TLS and spatial-stromal associations are NEN-specific; most cytokine and checkpoint data remain observational (16, 17, 65, 67–70, 72–76, 108).	Spatial transcriptome/proteome with matched blood and outcome data; functional perturbation in immune-competent models.
Hypoxia, vasculature, and neural routes	Abnormal angiogenesis and regional hypoxia select invasive states; vascular and perineural invasion provide anatomical routes for dissemination.	Angiogenic drivers and adverse pathology are documented, but endocrine phenotype is rarely tested as an independent modifier (17, 95–100, 102–107).	Co-register perfusion imaging, spatial hypoxia markers, vessel/nerve topology, and clone-specific dissemination.
Microbiome and host metabolism	Microbial metabolites may alter barrier integrity, systemic immunity, and mTOR/NF-κB-related signaling.	Associations occur across midgut, rectal, and pituitary NENs; causality, site transferability, and functional-status specificity are low-confidence (56, 85–94, 122–124).	Longitudinal diet/drug-controlled cohorts, paired tissue-stool-plasma sampling, and metabolite or microbial perturbation.
Therapy selection and recurrence	Treatment eliminates sensitive clones while receptor state, DNA repair, metabolism, stroma, and immune context permit residual disease and divergent relapse.	Randomized trials define treatment activity, whereas multi-omics predictors and organoid response signatures remain exploratory (71, 84, 101, 111–121, 125).	Locked predictive signature, external validation, prospective treatment-interaction test, and demonstration of clinical utility.

The framework yields falsifiable predictions. After matching for site, grade, stage, and treatment, an integrated state should improve out-of-sample recurrence prediction beyond clinicopathological variables; perturbing a nominated mediator should shift both its downstream omics module and invasive phenotype; and serial primary, metastatic, and relapse samples should show the predicted state transition or clonal selection. Failure of these tests would indicate that the cross-omic association is a passenger, a tissue-composition effect, or a consequence of progression.

### 7.2 Computational integration and systems biology

The integration method should follow the biological question. Early integration concatenates normalized features but is vulnerable to scale imbalance, missing modalities, and domination by the largest assay. Late integration combines modality-specific predictions and tolerates missing layers but may lose cross-layer interactions. Intermediate methods offer a useful compromise: iCluster and MOFA/MOFA+ learn shared latent factors; DIABLO selects correlated multi-omics features associated with a supervised outcome; and similarity network fusion combines patient-similarity graphs for subtype discovery (126–131). These approaches should be compared against single-modality and clinicopathological baselines, not only against one another.

Systems biology can then map latent factors to mechanism. Ligand-receptor analysis, transcription-factor and pathway activity inference, causal or Bayesian networks, and knowledge graphs can connect a genomic alteration to chromatin state, signaling, metabolism, cell-cell communication, ECM remodeling, and drug response. Pathway-constrained models are preferable to unconstrained feature lists when sample size is limited, provided that the prior network is not treated as proof of directionality (132, 133). Integration across patients should also preserve spatial and longitudinal structure rather than average primary, metastatic, pretreatment, and post-treatment samples into one state.

Rare-disease cohorts make artificial intelligence especially prone to optimism. Feature selection, imputation, batch correction, and hyperparameter tuning must occur within nested training folds; patients rather than samples should define data splits; and an independent institution should test the locked pipeline. Site, grade, stage, functionality, and treatment need explicit modeling to prevent shortcut learning. Calibration, uncertainty, missing-modality performance, and biological interpretability should be reported alongside discrimination. Federated or consortium analyses may expand sample size, but only after common data dictionaries, assay controls, and outcome definitions are established.

### 7.3 Translational technologies: promise and implementation barriers

Single-cell and spatial multi-omics can distinguish malignant lineage programs from immune, fibroblast, endothelial, and neural niches and can localize resistant states at invasive fronts (17, 84, 125, 134, 135). Current barriers include fresh-tissue requirements for some assays, dissociation and capture bias, limited field of view or spatial resolution, FFPE-related degradation, high cost, cross-platform batch effects, and difficult validation of rare cell states. Clinical feasibility will require targeted panels or multiplex assays derived from discovery data, with predefined regions, cell-state definitions, and quality controls.

Liquid biopsy offers longitudinal sampling but different analytes answer different questions. Tumor-specific mutations and copy-number changes are detectable in cell-free DNA from some metastatic pancreatic NETs (136), while multigene blood-transcript assays have shown diagnostic and monitoring signals in GEP and other NET cohorts (137, 138). Limitations include low tumor fraction in indolent or low-burden disease, variable shedding by site, clonal hematopoiesis for DNA assays, RNA and pre-analytical instability, non-equivalent assay versions and cutoffs, and case-mix-dependent specificity. Before routine use, a locked assay must show incremental value over imaging and pathology and demonstrate that acting on the result improves an outcome.

Patient-derived organoids can test drug response and generate perturbational data (84, 120), but establishment efficiency, clonal selection, loss of immune, vascular, neural, and stromal compartments, and turnaround time can limit representativeness. Co-culture or immune-competent models improve context but reduce standardization. Radiomics is more scalable and can sample whole-tumor heterogeneity (25, 26, 112, 121), yet scanner, acquisition, reconstruction, segmentation, and temporal variability threaten transportability. Multimodal models should therefore be tested across vendors and institutions and should retain a clinically usable fallback when one modality is unavailable.

### 7.4 Biomarker validation and clinical implementation

A multi-omics biomarker should pass four gates. Discovery defines a biologically coherent, parsimonious signature. Analytical validation locks specimen handling, assay precision, normalization, missing-data rules, and quality thresholds. Clinical validation uses prespecified cutoffs and endpoints in independent, multi-institutional cohorts and compares discrimination, calibration, and decision value with grade, stage, burden, and receptor imaging. Clinical-utility evaluation then tests whether biomarker-guided management improves patient outcome, reduces unnecessary procedures, or is cost-effective. REMARK and TRIPOD+AI provide reporting frameworks for prognostic biomarkers and prediction models (139, 140).

Reproducibility requires versioned code and assay protocols, harmonized pathology and outcome definitions, deposited metadata where consent permits, and replication across geographic and referral settings. A signature should be frozen before external validation, and its intended use must be explicit: diagnosis, recurrence risk, treatment selection, response monitoring, or minimal residual disease. Turnaround time, tissue consumption, cost, regulatory quality control, model updating, and clinician interpretation are part of validity rather than downstream administrative details. Without a linked decision and prospective utility test, a statistically significant multi-omics signature remains a research correlate.

### 7.5 Evidence boundaries and priority research questions

The mechanistic literature is dominated by pancreatic NETs. Evidence from duodenal or small-intestinal NETs, rectal NETs, bronchopulmonary and thymic NENs, pituitary NETs, and pheochromocytoma/paraganglioma is substantially thinner and reflects distinct cells of origin, exposures, metastatic routes, and definitions of functionality (60–63, 85–90, 141). The broad NEN framework should therefore be used to transfer testable hypotheses, not to imply that a pancreatic molecular program has already been validated across organs.

Priority questions are whether hormone secretion causally alters invasion or mainly changes detection; whether functionality is stable during progression and therapy; which primary-tumor states seed liver, nodal, bone, or other niches; which immune-stromal programs sustain residual disease; and which integrated biomarkers predict treatment interaction rather than prognosis alone. The most informative design is a prospective, multi-site cohort with harmonized functional annotation, central pathology, multi-region primary and metastasis sampling, serial blood and imaging, common treatment and outcome metadata, and a prespecified external validation cohort. Functional and non-functional tumors should be compared within site and grade, while NECs remain a separate biological stratum.

## 8 Conclusion

The evidence favors a conditional spectrum rather than a binary metastatic program. Functionality influences symptoms, diagnostic timing, peri-treatment physiology, and sometimes lineage composition, but metastatic competence emerges from coupled genome-epigenome states, adaptive signaling and metabolism, and permissive immune-stromal-vascular niches. The relative contribution of each layer varies by primary site, differentiation, grade, stage, and treatment history.

The immediate translational goal is not a universal functional-versus-non-functional classifier. It is a reproducible, site-specific model that adds calibrated value beyond pathology, stage, somatostatin-receptor imaging, and disease tempo, and that points to an actionable treatment or surveillance decision. Achieving this goal requires longitudinal, pathologically matched cohorts; external and prospective validation; transparent computational practice; and explicit recognition that current multi-omics evidence is dominated by pancreatic NETs. Under those conditions, the spectrum framework becomes a testable strategy for recurrence prediction and therapy selection rather than a descriptive taxonomy.

## Glossary

AI: Artificial Intelligence
ALT: Alternative Lengthening of Telomeres
ACSS2: Acyl-CoA Synthetase Short-Chain Family Member 2
aNENs: advanced/high-grade neuroendocrine neoplasms
ANGPT2: Angiopoietin-2
APOE: Apolipoprotein E
ATAC-seq: Assay for Transposase-Accessible Chromatin with high-throughput sequencing
ATRX: Alpha Thalassemia/Mental Retardation Syndrome X-Linked
B7-H3: B7 Homolog 3
CAFs: Cancer-Associated Fibroblasts
CD3: Cluster of Differentiation 3
CD8: Cluster of Differentiation 8
CDKN1B: Cyclin-Dependent Kinase Inhibitor 1B
CSF-1R: Colony-Stimulating Factor 1 Receptor
cfDNA: cell-free DNA
ctDNA: circulating tumor DNA
CT: Computed Tomography
CTLA-4: Cytotoxic T-Lymphocyte-Associated Protein 4
CX3CR1: C-X3-C Motif Chemokine Receptor 1
CXCL8: C-X-C motif chemokine ligand 8 (IL-8)
DAXX: Death-Domain Associated Protein
DFS: Disease-Free Survival
DIABLO: Data Integration Analysis for Biomarker discovery using Latent cOmponents
DNMT3B: DNA (Cytosine-5)-Methyltransferase 3B
ECM: Extracellular Matrix
EMT: Epithelial-Mesenchymal Transition
ERVs: Endogenous Retroviruses
f-NETs: functional neuroendocrine tumors
FGFR1: Fibroblast Growth Factor Receptor 1
FOXO: Forkhead box O (transcription factor family)
GEP-NETs: Gastroenteropancreatic neuroendocrine tumors
GH-PitNETs: Growth Hormone-secreting pituitary neuroendocrine tumors
HDACs: Histone Deacetylases
HHLA2: Human Endogenous Retrovirus-H Long Terminal Repeat-Associating Protein 2
HRAS: Harvey Rat Sarcoma viral oncogene homolog
IL-6: Interleukin-6
IL-8: Interleukin-8
IL-10: Interleukin-10
IL-17: Interleukin-17
IL-18: Interleukin-18
Ki-67: Ki-67 proliferation index
MAPK: Mitogen-Activated Protein Kinase
MEN1: Multiple Endocrine Neoplasia type 1
ML: Machine Learning
MOFA: Multi-Omics Factor Analysis
MRI: Magnetic Resonance Imaging
mTOR: mechanistic target of rapamycin
MYC: MYC proto-oncogene
NECs: Neuroendocrine Carcinomas
NENs: neuroendocrine neoplasms
NETs: neuroendocrine tumors
NF-κB: Nuclear Factor kappa-B
nf-NETs: non-functional neuroendocrine tumors
NF-PanNETs: non-functional pancreatic neuroendocrine tumors
nf-PitNETs: non-functional pituitary neuroendocrine tumors
NR5A1: Nuclear Receptor Subfamily 5 Group A Member 1
OXPHOS: Oxidative Phosphorylation
PanNETs: Pancreatic neuroendocrine tumors
PD-L1: Programmed Death-Ligand 1
PDGFB: Platelet-Derived Growth Factor Subunit B
PGC-1α: Peroxisome Proliferator-Activated Receptor Gamma Coactivator 1-alpha
PI3K: Phosphoinositide 3-Kinase
PIK3CA: Phosphatidylinositol-4, 5-Bisphosphate 3-Kinase Catalytic Subunit Alpha
PRC1/2: Polycomb Repressive Complex 1/2
PRRT: Peptide Receptor Radionuclide Therapy
PTEN: Phosphatase and Tensin Homolog
RABL6A: RAB-Like 6A
RAS: Rat sarcoma (RAS family of small GTPases)
RAS/MAPK: RAS/Mitogen-Activated Protein Kinase pathway
RB1: Retinoblastoma 1
rNETs: rectal neuroendocrine tumors
SCARB1: Scavenger Receptor Class B Member 1
SCFAs: Short-Chain Fatty Acids
SCNCs: Small-Cell Neuroendocrine Cancers
SCLC: Small Cell Lung Cancer
SII: Systemic Immune-Inflammation Index
SNF: Similarity Network Fusion
SSTR2: Somatostatin Receptor 2
STAT3: Signal Transducer and Activator of Transcription 3
TAMs: Tumor-Associated Macrophages
TGF-β: Transforming Growth Factor-beta
TLS: Tertiary Lymphoid Structure
TRIPOD+AI: Transparent Reporting of a multivariable prediction model for Individual Prognosis Or Diagnosis plus Artificial Intelligence
TIM-3: T-cell Immunoglobulin and Mucin-domain containing-3
TIE2: Tyrosine kinase with immunoglobulin-like and EGF-like domains 2 (TEK)
TME: Tumor Microenvironment
TNF-α: Tumor Necrosis Factor-alpha
TP53: Tumor Protein p53
Trp53: Transformation-related protein 53 (mouse homolog of TP53)
TSC2: Tuberous Sclerosis Complex 2
T-NEN: Thymic Neuroendocrine Neoplasm
VEGF: Vascular Endothelial Growth Factor
VEGFR: Vascular Endothelial Growth Factor Receptor
VASH-1: Vasohibin-1
Wnt: Wingless-related integration site

## References

[1] Rindi G Mete O Uccella S Basturk O La Rosa S Brosens LAA . Overview of the 2022 WHO classification of neuroendocrine neoplasms. Endocr Pathol. (2022) 33:115–54. doi: 10.1007/s12022-022-09708-2 35294740

[2] Hofland J Falconi M Christ E Castaño JP Faggiano A Lamarca A . European Neuroendocrine Tumor Society 2023 guidance paper for functioning pancreatic neuroendocrine tumour syndromes. J Neuroendocrinol. (2023) 35:e13318. doi: 10.1111/jne.13318 37578384

[3] Magi L Marasco M Rinzivillo M Fazio N . Management of functional pancreatic neuroendocrine neoplasms. Curr Treat Options Oncol. (2023) 24:725–41. doi: 10.1007/s11864-023-01085-0 37103745 PMC10272249

[4] Kos-Kudła B Castaño JP Denecke T Grande E Kjaer A Koumarianou A . European Neuroendocrine Tumour Society (ENETS) 2023 guidance paper for nonfunctioning pancreatic neuroendocrine tumours. J Neuroendocrinol. (2023) 35:e13343. doi: 10.1111/jne.13343 37877341

[5] Gorelik M Ahmad M Grossman D . Nonfunctioning incidental pancreatic neuroendocrine tumors: who, when, and how to treat? Surg Clin North Am. (2018) 98:157–67. doi: 10.1016/j.suc.2017.09.014 29191272

[6] Pellat A Barat M Cottereau AS Coriat R . Well-differentiated neuroendocrine tumors of the digestive tract: focus on pancreatic neuroendocrine tumors. Bull Cancer. (2023) 110:955–67. doi: 10.1016/j.bulcan.2023.03.001 36935319

[7] Chen Z Lin S Liang F Hou Z Yang Y Huang H . The prognostic and therapeutic value of the tumor microenvironment and immune checkpoints in pancreatic neuroendocrine neoplasms. Sci Rep. (2024) 14:24669. doi: 10.1038/s41598-024-75882-4 39433799 PMC11494001

[8] Yan J Yu S Jia C Li M Chen J . Molecular subtyping in pancreatic neuroendocrine neoplasms: New insights into clinical, pathological unmet needs and challenges. Biochim Biophys Acta Rev Cancer. (2020) 1874:188367. doi: 10.1016/j.bbcan.2020.188367 32339609

[9] van Riet J van de Werken HJG Cuppen E Eskens FALM Tesselaar MET van Veenendaal LM . The genomic landscape of 85 advanced neuroendocrine neoplasms reveals subtype-heterogeneity and potential therapeutic targets. Nat Commun. (2021) 12:4612. doi: 10.1038/s41467-021-24812-3 34326338 PMC8322054

[10] Scarpa A Chang DK Nones K Corbo V Patch AM Bailey P . Whole-genome landscape of pancreatic neuroendocrine tumours. Nature. (2017) 543:65–71. doi: 10.1038/nature21063 28199314

[11] Figueira ERR Montagnini AL Okubo J Fernandes AGV Pereira MA Ribeiro Jr U . Non-functioning sporadic pancreatic neuroendocrine tumor is an independent risk factor for recurrence after surgical treatment. Arq Bras Cir Dig. (2024) 37:e1857. doi: 10.1590/0102-6720202400063e1857 39841762 PMC11745474

[12] Ventin M Arya S Zhang L Gangi A Fernandez del-Castillo C Qadan M . Recurrence in patients with lymph node-negative pancreatic neuroendocrine tumors. JAMA Surg. (2026) 161:142–51. doi: 10.1001/jamasurg.2025.5401 41405980 PMC12712833

[13] Ugras N Hasdemir S Yerci O Tasar P Dundar HZ Macunluoglu AC . Do morphologic characteristics play a role in nodal metastatic progression of well-differentiated pancreatic neuroendocrine tumors? Tumori. (2021) 107:80–5. doi: 10.1177/0300891620941921 32705937

[14] Tobias J Clarke CN Gangi A Keutgen XM . The Landmark Series: Surgical management of functioning and non-functioning pancreatic neuroendocrine tumors. Ann Surg Oncol. (2025) 32:4720–8. doi: 10.1245/s10434-025-17390-x 40319207 PMC12130066

[15] Ma M Gu W Liang Y Han X Zhang M Xu M . AA novel model for predicting postoperative liver metastasis in R0 resected pancreatic neuroendocrine tumors: integrating computational pathology and deep learning-radiomics. J Transl Med. (2024) 22:768. doi: 10.1186/s12967-024-05449-4 39143624 PMC11323380

[16] Zhang W-H Wang W-Q Han X Gao H-L Xu S-S Li S . Infiltrating pattern and prognostic value of tertiary lymphoid structures in resected non-functional pancreatic neuroendocrine tumors. J Immunother Cancer. (2020) 8:e001188. doi: 10.1136/jitc-2020-001188 33055204 PMC7559054

[17] Ye Z Li Q Hu Y Hu H Xu J Guo M . The stromal microenvironment endows pancreatic neuroendocrine tumors with spatially specific invasive and metastatic phenotypes. Cancer Lett. (2024) 588:216769. doi: 10.1016/j.canlet.2024.216769 38438098

[18] Mohindroo C Baydogan S Agarwal P Wright RD Prakash LR Mork ME . Germline testing identifies pathogenic/likely pathogenic variants in patients with pancreatic neuroendocrine tumors. Cancer Prev Res (Phila). (2024) 17:335–42. doi: 10.1158/1940-6207.CAPR-23-0483 38662083

[19] Matkar S Thiel A Hua X . Menin: a scaffold protein that controls gene expression and cell signaling. Trends Biochem Sci. (2013) 38:394–402. doi: 10.1016/j.tibs.2013.05.005 23850066 PMC3741089

[20] Pipinikas CP Berner AM Sposito T Thirlwell C . The evolving (epi)genetic landscape of pancreatic neuroendocrine tumours. Endocr Relat Cancer. (2019) 26:R519–44. doi: 10.1530/erc-19-0175 31252410

[21] Tan Q Liu L Liu X Tan C Wang X . Prognosis of small pancreatic neuroendocrine neoplasms: Functionality matters. Am J Surg. (2025) 246:116302. doi: 10.1016/j.amjsurg.2025.116302 40140248

[22] Mattiolo P Bevere M Mafficini A Verschuur AV Calicchia M Hackeng WM . Glucagon-producing pancreatic neuroendocrine tumors (glucagonomas) are enriched in aggressive neoplasms with ARX and PDX1 co-expression, DAXX/ATRX mutations, and alternative lengthening of telomeres. Endocr Pathol. (2024) 35:354–61. doi: 10.1007/s12022-024-09826-z 39331358 PMC11659356

[23] Nießen A Schimmack S Lewosinska M Hinz U Bechtiger FA Hackert T . Lymph node metastases and recurrence in pancreatic neuroendocrine neoplasms. Surgery. (2022) 172:1791–9. doi: 10.1016/j.surg.2022.08.020 36180252

[24] Lu Z Li T Liu C Zheng Y Song J . Development and validation of a survival prediction model and risk stratification for pancreatic neuroendocrine neoplasms. J Endocrinol Invest. (2023) 46:927–37. doi: 10.1007/s40618-022-01956-7 36394822

[25] Zhu HB Zhu HT Jiang L Nie P Hu J Tang W . Radiomics analysis from magnetic resonance imaging in predicting the grade of nonfunctioning pancreatic neuroendocrine tumors: a multicenter study. Eur Radiol. (2024) 34:90–102. doi: 10.1007/s00330-023-09957-7 37552258 PMC10791720

[26] Bian Y Jiang H Ma C Wang L Zheng J Jin G . CT-based radiomics score for distinguishing between grade 1 and grade 2 nonfunctioning pancreatic neuroendocrine tumors. AJR Am J Roentgenol. (2020) 215:852–63. doi: 10.2214/ajr.19.22123 32755201

[27] Chen G Liu L Tan C Tan Q Chen Y An X . Prognostic significance of systemic immune-inflammation index in patients with nonfunction pancreatic neuroendocrine tumor undergoing surgical resection. Cancer Med. (2024) 13:e7114. doi: 10.1002/cam4.7114 38553949 PMC10980930

[28] Chinese Multidisciplinary Expert Consensus on the Rational Use of Surufatinib in Clinical Practice-Editorial Board Group . Chinese multidisciplinary expert consensus on the rational use of surufatinib in clinical practice (2024 edition). Zhonghua Zhong Liu Za Zhi. (2024) 46:930–9. doi: 10.3760/cma.j.cn112152-20240524-00216 39414593

[29] Andreasi V Partelli S Muffatti F Manzoni MF Capurso G Falconi M . Update on gastroenteropancreatic neuroendocrine tumors. Dig Liver Dis. (2021) 53:171–82. doi: 10.1016/j.dld.2020.08.031 32912771

[30] Lu X Yan S Koral KA Chen Z . Surufatinib for the treatment of advanced extrapancreatic neuroendocrine tumors. Expert Rev Anticancer Ther. (2021) 21:917–26. doi: 10.1080/14737140.2021.1944110 34142932

[31] Jiao Y Shi C Edil BH de Wilde RF Klimstra DS Maitra A . DAXX/ATRX, MEN1, and mTOR pathway genes are frequently altered in pancreatic neuroendocrine tumors. Science. (2011) 331:1199–203. doi: 10.1126/science.1200609 21252315 PMC3144496

[32] Marinoni I Schmitt Kurrer A Vassella E Dettmer M Rudolph T Banz V . Loss of DAXX and ATRX are associated with chromosome instability and reduced survival of patients with pancreatic neuroendocrine tumors. Gastroenterology. (2014) 146:453–460.e5. doi: 10.1053/j.gastro.2013.10.020 24148618

[33] Chan CS Laddha SV Lewis PW Koletsky MS Robzyk K Da Silva E . ATRX, DAXX or MEN1 mutant pancreatic neuroendocrine tumors are a distinct alpha-cell signature subgroup. Nat Commun. (2018) 9:4158. doi: 10.1038/s41467-018-06498-2 30315258 PMC6185985

[34] Yachida S Vakiani E White CM Zhong Y Saunders T Morgan R . Small cell and large cell neuroendocrine carcinomas of the pancreas are genetically similar and distinct from well-differentiated pancreatic neuroendocrine tumors. Am J Surg Pathol. (2012) 36:173–84. doi: 10.1097/pas.0b013e3182417d36 22251937 PMC3261427

[35] Vousden KH Lane DP . p53 in health and disease. Nat Rev Mol Cell Biol. (2007) 8:275–83. doi: 10.1038/nrm2147 17380161

[36] Derks JL Leblay N Thunnissen E van Suylen RJ den Bakker M Groen HJM . Molecular subtypes of pulmonary large-cell neuroendocrine carcinoma predict chemotherapy treatment outcome. Clin Cancer Res. (2018) 24:33–42. doi: 10.1158/1078-0432.ccr-17-1921 29066508

[37] Arakelyan J Zohrabyan D Philip PA . Molecular profile of pancreatic neuroendocrine neoplasms (PanNENs): opportunities for personalized therapies. Cancer. (2021) 127:345–53. doi: 10.1002/cncr.33354 33270905

[38] Peifer M Fernández-Cuesta L Sos ML George J Seidel D Kasper LH . Integrative genome analyses identify key somatic driver mutations of small-cell lung cancer. Nat Genet. (2012) 44:1104–10. doi: 10.1038/ng.2396 22941188 PMC4915822

[39] Levine AJ Oren M . The first 30 years of p53: growing ever more complex. Nat Rev Cancer. (2009) 9:749–58. doi: 10.1038/nrc2723 19776744 PMC2771725

[40] Burkhart DL Sage J . Cellular mechanisms of tumour suppression by the retinoblastoma gene. Nat Rev Cancer. (2008) 8:671–82. doi: 10.1038/nrc2399 18650841 PMC6996492

[41] Lakis V Lawlor RT Newell F Patch A-M Mafficini A Sadanandam A . DNA methylation patterns identify subgroups of pancreatic neuroendocrine tumors with clinical association. Commun Biol. (2021) 4:155. doi: 10.1038/s42003-020-01469-0 33536587 PMC7859232

[42] Di Domenico A Pipinikas CP Maire RS Bräutigam K Simillion C Dettmer MS . Epigenetic landscape of pancreatic neuroendocrine tumours reveals distinct cells of origin and means of tumour progression. Commun Biol. (2020) 3:740. doi: 10.1038/s42003-020-01479-y 33288854 PMC7721725

[43] Hackeng WM Brosens LAA Kim JY O'Sullivan R Sung Y-N Liu T-C . Non-functional pancreatic neuroendocrine tumours: ATRX/DAXX and alternative lengthening of telomeres (ALT) are prognostically independent from ARX/PDX1 expression and tumour size. Gut. (2022) 71:961–73. doi: 10.1136/gutjnl-2020-322595 33849943 PMC8511349

[44] Wasylishen AR Sun C Moyer SM Qi Y Chau GP Aryal NK . Daxx maintains endogenous retroviral silencing and restricts cellular plasticity in vivo. Sci Adv. (2020) 6:eaba8415. doi: 10.1126/sciadv.aba8415 32821827 PMC7406367

[45] Wang D Di X Gao F Li G Lin L He S . Epigenomic heterogeneity of non-functional pancreatic neuroendocrine tumors uncovered by single nucleus and spatial atac profiling. bioRxiv Preprint. (2025), 2025.09.11.675640. doi: 10.1101/2025.09.11.675640

[46] Andersson E Arvidsson Y Swärd C Hofving T Wängberg B Kristiansson E . Expression profiling of small intestinal neuroendocrine tumors identifies subgroups with clinical relevance, prognostic markers and therapeutic targets. Mod Pathol. (2016) 29:616–29. doi: 10.1038/modpathol.2016.48 26965582

[47] Madigan JP Andrews SG Farrell RB Forsythe SD Sharma R Ceribelli M . Multiple epigenetic mechanisms functionally cooperate to silence expression of somatostatin receptor type 2 in pancreatic neuroendocrine tumors. bioRxiv Preprint. (2025), 2025.09.23.677935. doi: 10.1101/2025.09.23.677935

[48] Klomp MJ Dalm SU de Jong M Feelders RA Hofland J Hofland LJ . Epigenetic regulation of somatostatin and somatostatin receptors in neuroendocrine tumors and other types of cancer. Rev Endocr Metab Disord. (2021) 22:495–510. doi: 10.1007/s11154-020-09607-z 33085037 PMC8346415

[49] Rindi G Wiedenmann B . Neuroendocrine neoplasia of the gastrointestinal tract revisited: towards precision medicine. Nat Rev Endocrinol. (2020) 16:590–607. doi: 10.1038/s41574-020-0391-3 32839579

[50] Falletta S Partelli S Rubini C Nann D Doria A Marinoni I . mTOR inhibitors response and mTOR pathway in pancreatic neuroendocrine tumors. Endocr Relat Cancer. (2016) 23:883–91. doi: 10.1530/erc-16-0329 27697900

[51] Zitzmann K De Toni EN Brand S Göke B Meinecke J Spöttl G . The novel mTOR inhibitor RAD001 (everolimus) induces antiproliferative effects in human pancreatic neuroendocrine tumor cells. Neuroendocrinology. (2007) 85:54–60. doi: 10.1159/000100057 17310129

[52] Pavel M Öberg K Falconi M Krenning EP Sundin A Perren A . Gastroenteropancreatic neuroendocrine neoplasms: ESMO Clinical Practice Guidelines for diagnosis, treatment and follow-up. Ann Oncol. (2020) 31:844–60. doi: 10.1016/j.annonc.2020.03.304 32272208

[53] O'Reilly KE Rojo F She QB Solit D Mills GB Smith D . mTOR inhibition induces upstream receptor tyrosine kinase signaling and activates Akt. Cancer Res. (2006) 66:1500–8. doi: 10.1158/0008-5472.CAN-05-2925 PMC3193604 16452206

[54] Carracedo A Ma L Teruya-Feldstein J Rojo F Salmena L Alimonti A . Inhibition of mTORC1 leads to MAPK pathway activation through a PI3K-dependent feedback loop in human cancer. J Clin Invest. (2008) 118:3065–74. doi: 10.1172/jci34739 18725988 PMC2518073

[55] Gu D Ye M Zhu G Bai J Chen J Yan L . Hypoxia upregulating ACSS2 enhances lipid metabolism reprogramming through HMGCS1-mediated PI3K/AKT/mTOR pathway to promote the progression of pancreatic neuroendocrine neoplasms. J Transl Med. (2024) 22:93. doi: 10.1186/s12967-024-04870-z 38263056 PMC10804556

[56] Ye Z Chen H Ji S Hu Y Lou X Zhang W . MEN1 promotes ferroptosis by inhibiting the mTOR-SCD1 axis in pancreatic neuroendocrine tumors. Acta Biochim Biophys Sin (Shanghai). (2022) 54:1599–609. doi: 10.3724/abbs.2022162 36604142 PMC9828289

[57] Gui S Yu W Xie J Peng L Xiong Y Song Z . SLC7A11 promotes EMT and metastasis in invasive pituitary neuroendocrine tumors by activating the PI3K/AKT signaling pathway. Endocr Connect. (2024) 13:e240097. doi: 10.1530/ec-24-0097 38722255 PMC11227052

[58] Saleh Z Moccia MC Ladd Z Joneja U Li Y Spitz F . Pancreatic neuroendocrine tumors: signaling pathways and epigenetic regulation. Int J Mol Sci. (2024) 25:1331. doi: 10.3390/ijms25021331 38279330 PMC10816436

[59] Pan J Bao Q Enders G . The altered metabolic molecular signatures contribute to the RAD001 resistance in gastric neuroendocrine tumor. Front Oncol. (2020) 10:546. doi: 10.3389/fonc.2020.00546 32373532 PMC7186336

[60] Rico K Duan S Pandey RL Chen Y Chakrabarti JT Starr J . Genome analysis identifies differences in the transcriptional targets of duodenal versus pancreatic neuroendocrine tumours. BMJ Open Gastroenterol. (2021) 8:e000765. doi: 10.1136/bmjgast-2021-000765 34750164 PMC8576490

[61] Liu M Hu W Zhang Y Zhang N Chen L Lin Y . Clinical implications of immune checkpoint markers and immune infiltrates in patients with thymic neuroendocrine neoplasms. Front Oncol. (2022) 12:917743. doi: 10.3389/fonc.2022.917743 36203421 PMC9531766

[62] Ghosal S Hadrava Vanova K Uher O Das S Patel M Meuter L . Immune signature of pheochromocytoma and paraganglioma in context of neuroendocrine neoplasms associated with prognosis. Endocrine. (2023) 79:171–9. doi: 10.1007/s12020-022-03218-1 36370152 PMC10683554

[63] Lin S Dai Y Han C Han T Zhao L Wu R . Single-cell transcriptomics reveal distinct immune-infiltrating phenotypes and macrophage-tumor interaction axes among different lineages of pituitary neuroendocrine tumors. Genome Med. (2024) 16:60. doi: 10.1186/s13073-024-01325-4 38658971 PMC11040908

[64] Zhang J Zhang H Zhang L Li D Qi M Zhang L . Single-cell transcriptome identifies drug-resistance signature and immunosuppressive microenvironment in metastatic small cell lung cancer. Adv Genet (Hoboken). (2022) 3:2100060. doi: 10.1002/ggn2.202100060 36618022 PMC9744506

[65] Busse A Mochmann LH Spenke C Arsenic R Briest F Jöhrens K . Immunoprofiling in neuroendocrine neoplasms unveil immunosuppressive microenvironment. Cancers (Basel). (2020) 12:3448. doi: 10.3390/cancers12113448 33228231 PMC7699546

[66] Suteau V Collin A Menei P Rodien P Rousselet M-C Briet C . Expression of programmed death-ligand 1 (PD-L1) in human pituitary neuroendocrine tumors. Cancer Immunol Immunother. (2020) 69:2053–61. doi: 10.1007/s00262-020-02611-x 32445029 PMC11027687

[67] Mo S Zong L Chen X Ban X Li M Lu Z . Expression and prognostic value of B7 family immune checkpoints in pancreatic neuroendocrine tumors. Arch Pathol Lab Med. (2023) 147:193–201. doi: 10.5858/arpa.2021-0377-oa 35671167

[68] Copur MS Vargas L Wedel W Merani S Cushman-Vokoun A Drincic A . Pancreatic neuroendocrine tumor with humoral hypercalcemia and high tumor PD-L1 score. Oncol (Williston Park). (2020) 34:548–52. doi: 10.46883/onc.2020.3412.0548 33395496

[69] Yuan Z Gardiner JC Maggi EC Huang S Adem A Bagdasarov S . B7 immune-checkpoints as targets for the treatment of neuroendocrine tumors. Endocr Relat Cancer. (2021) 28:135–49. doi: 10.1530/erc-20-0337 33410766 PMC8486311

[70] Gruetzmacher C Pessoa BS Craveiro FL Nakaguma M Trarbach EB Batista RL . HHLA2: a potential biomarker and therapeutic target in endocrine-related cancer. Endocr Oncol. (2025) 5:e240034. doi: 10.1530/eo-24-0034 40255615 PMC12007912

[71] Halperin DM Liu S Dasari A Fogelman D Bhosale P Mahvash A . Assessment of clinical response following atezolizumab and bevacizumab treatment in patients with neuroendocrine tumors. JAMA Oncol. (2022) 8:904–9. doi: 10.1001/jamaoncol.2022.0212 35389428 PMC8990358

[72] Korkaya H Liu S Wicha MS . Regulation of cancer stem cells by cytokine networks: attacking cancer's inflammatory roots. Clin Cancer Res. (2011) 17:6125–9. doi: 10.1158/1078-0432.ccr-10-2743 21685479 PMC3312242

[73] Yu H Pardoll D Jove R . STATs in cancer inflammation and immunity: a leading role for STAT3. Nat Rev Cancer. (2009) 9:798–809. doi: 10.1038/nrc2734 19851315 PMC4856025

[74] Matsuzaki H Komohara Y Yano H Fujiwara Y Kai K Yamada R . Macrophage colony-stimulating factor potentially induces recruitment and maturation of macrophages in recurrent pituitary neuroendocrine tumors. Microbiol Immunol. (2023) 67:90–8. doi: 10.1111/1348-0421.13041 36461910

[75] Geisler L Hellberg T Lambrecht J Jann H Knorr J Eschrich J . Inflammatory cytokines associated with diagnosis, tumor grade and prognosis in patients with neuroendocrine tumors. J Clin Med. (2022) 11:6191. doi: 10.3390/jcm11206191 36294509 PMC9604855

[76] Zhang Y Manouchehri Doulabi E Herre M Cedervall J Qiao Q Miao Z . Platelet-derived PDGFB promotes recruitment of cancer-associated fibroblasts, deposition of extracellular matrix and TGFβ signaling in the tumor microenvironment. Cancers (Basel). (2022) 14:1947. doi: 10.3390/cancers14081947 35454853 PMC9024906

[77] Jannin A Dessein A-F Cao DD Vantyghem M-C Chevalier B Van Seuningen I . Metabolism of pancreatic neuroendocrine tumors: what can omics tell us? Front Endocrinol (Lausanne). (2023) 14:1248575. doi: 10.3389/fendo.2023.1248575 37908747 PMC10613989

[78] Hu C Chen L Ding Y Ye M Tang Q . Metabolic changes in neuroendocrine neoplasms. Cell Mol Life Sci. (2025) 82:205. doi: 10.1007/s00018-025-05656-2 40377669 PMC12084448

[79] Ozbudak IH Shilo K Tavora F Rassaei N Chu W-S Fukuoka J . Glucose transporter-1 in pulmonary neuroendocrine carcinomas: expression and survival analysis. Mod Pathol. (2009) 22:633–8. doi: 10.1038/modpathol.2009.6 19234439 PMC3616507

[80] Varuzhanyan G Chen C-C Freeland J He T Tran W Song K . PGC-1α drives small cell neuroendocrine cancer progression towards an ASCL1-expressing subtype with increased mitochondrial capacity. Proc. Natl. Acad. Sci. USA. (2024) 121:e2416882121. doi: 10.1073/pnas.2416882121 39589879 PMC11626175

[81] Ye M Lu F Chen J Yu P Xu Y He N . Orlistat induces ferroptosis in pancreatic neuroendocrine tumors by inactivating the MAPK pathway. J Cancer. (2023) 14:1458–69. doi: 10.7150/jca.83118 37283794 PMC10240670

[82] Lou X Shi Y Qin Y Zhang W Ye Z Wang F . Tumor-derived apolipoprotein E confers resistance to temozolomide in pancreatic neuroendocrine tumors. Cell Death Dis. (2026) 17:35. doi: 10.1038/s41419-025-08317-1 41390817 PMC12804833

[83] Wen S Li J Yang J Li B Li N Zhan X . Quantitative acetylomics revealed acetylation-mediated molecular pathway network changes in human nonfunctional pituitary neuroendocrine tumors. Front Endocrinol (Lausanne). (2021) 12:753606. doi: 10.3389/fendo.2021.753606 34712204 PMC8546192

[84] Ji S Cao L Gao J Du Y Ye Z Lou X . Proteogenomic characterization of non-functional pancreatic neuroendocrine tumors unravels clinically relevant subgroups. Cancer Cell. (2025) 43:776–796.e14. doi: 10.1016/j.ccell.2025.03.016 40185092

[85] Mulders MCF Audhoe AS van Koetsveld PM Feelders RA Hofland LJ de Herder WW . Midgut neuroendocrine tumor patients have a depleted gut microbiome with a discriminative signature. Eur J Cancer. (2024) 197:113472. doi: 10.1016/j.ejca.2023.113472 38100919

[86] Mohamed A Asa SL McCormick T Al-Shakhshir H Dasari A Mauricio R . The role of the microbiome in gastroentero-pancreatic neuroendocrine neoplasms. Curr Issues Mol Biol. (2022) 44:2015–28. doi: 10.3390/cimb44050136 35678665 PMC9164086

[87] El Tekle G Garrett WS . Bacteria in cancer initiation, promotion and progression. Nat Rev Cancer. (2023) 23:600–18. doi: 10.1038/s41568-023-00594-2 37400581

[88] Gao Y Zheng H Ye M Zhou G Chen J Qiang X . Characteristics and function of the gut microbiota in patients with rectal neuroendocrine tumors. J Cancer. (2025) 16:1040–50. doi: 10.7150/jca.103297 39895797 PMC11786048

[89] Liu J Ye Z Zhang Y Su W Liu J Chen T . Exploring the gut microbiome and serum metabolome interplay in non-functioning pituitary neuroendocrine tumors. Front Microbiol. (2025) 16:1541683. doi: 10.3389/fmicb.2025.1541683 40236482 PMC11997625

[90] Liu J Ye Z Zhang Y You S Sun S Lu L . Multi-omics study reveals gut microbiota dysbiosis and tryptophan metabolism alterations in GH-PitNET progression. Sci Rep. (2025) 15:24261. doi: 10.1038/s41598-025-07812-x 40624207 PMC12234824

[91] Zhang Z Li D Xie F Muhetaer G Zhang H . The cause-and-effect relationship between gut microbiota abundance and carcinoid syndrome: a bidirectional Mendelian randomization study. Front Microbiol. (2023) 14:1291699. doi: 10.3389/fmicb.2023.1291699 38188562 PMC10766758

[92] Johansen SU Goll R Nordborg A Vernstad K Jensen EPH Florholmen JR . Plasma levels of organic acids associated with the gut microbiome display significant alterations in neuroendocrine tumor patients. Neuroendocrinology. (2025) 115:283–94. doi: 10.1159/000543247 39715594

[93] Mann ER Lam YK Uhlig HH . Short-chain fatty acids: linking diet, the microbiome and immunity. Nat Rev Immunol. (2024) 24:577–95. doi: 10.1038/s41577-024-01014-8 38565643

[94] Li S Duan Y Luo S Zhou F Wu Q Lu Z . Short-chain fatty acids and cancer. Trends Cancer. (2025) 11:154–68. doi: 10.1016/j.trecan.2024.11.003 39638744

[95] Maharjan CK Umesalma S Kaemmer CA Muniz VP Bauchle C Mott SL . RABL6A promotes pancreatic neuroendocrine tumor angiogenesis and progression *in vivo*. Biomedicines. (2021) 9:633. doi: 10.3390/biomedicines9060633 34199469 PMC8228095

[96] Strzelczyk J Wójcik-Giertuga M Cuber P Biernacki K Kos-Kudła B Strzelczyk JK . Assessment of the concentration of endogenous factors regulating angiogenesis, VASH-1 and VEGF-A, in the blood serum of patients with neuroendocrine neoplasms. BioMed Res Int. (2022) 2022:9084393. doi: 10.1155/2022/9084393 35372578 PMC8966743

[97] Minaskan Karabid N Wiedemann T Gulde S Mohr H Segaran RC Geppert J . Angpt2/Tie2 autostimulatory loop controls tumorigenesis. EMBO Mol Med. (2022) 14:e14364. doi: 10.15252/emmm.202114364 35266635 PMC9081903

[98] Fujiwara R Ten H Chen H Jiang C-L Oyama K-I Onoda K . Cathepsin D inhibits angiogenesis in pituitary neuroendocrine tumors. Acta Histochem Cytochem. (2022) 55:203–11. doi: 10.1267/ahc.22-00098 36688139 PMC9840469

[99] Chen X Ke X Lu Y Miao H Duan L Yang H . The multifaceted roles of pituitary pericytes in health and disease. Endocrinology. (2025) 166:bqaf152. doi: 10.1210/endocr/bqaf152 41118149

[100] Luo X He J-Y Xu J Hu S-Y Mo B-H Shu Q-X . Vascular NRP2 triggers PNET angiogenesis by activating the SSH1-cofilin axis. Cell Biosci. (2020) 10:113. doi: 10.1186/s13578-020-00472-6 32983407 PMC7509939

[101] Chan JA Geyer S Zemla T Knopp MV Behr S Pulsipher S . Phase 3 trial of cabozantinib to treat advanced neuroendocrine tumors. N Engl J Med. (2025) 392:653–65. doi: 10.1056/nejmoa2403991 39282913 PMC11821447

[102] Zhou H Wang Y Guo C Li X Cui W Wang Z . Microscopic invasion of nerve is associated with aggressive behaviors in pancreatic neuroendocrine tumors. Front Oncol. (2021) 11:630316. doi: 10.3389/fonc.2021.630316 33718210 PMC7947608

[103] Shi C Chen W Davis R Morse MA . Venous invasion in pancreatic neuroendocrine tumors is independently associated with disease-free survival and overall survival. Am J Surg Pathol. (2023) 47:678–85. doi: 10.1097/pas.0000000000002038 37017316

[104] Iżycka-Świeszewska E Gulczyński J Sejda A Kitlińska J Galli S Rogowski W . Remarks on selected morphological aspects of cancer neuroscience: a microscopic photo review. Biomedicines. (2024) 12:2335. doi: 10.3390/biomedicines12102335 39457647 PMC11505290

[105] Sigorski D Gulczyński J Sejda A Rogowski W Iżycka-Świeszewska E . Investigation of neural microenvironment in prostate cancer in context of neural density, perineural invasion, and neuroendocrine profile of tumors. Front Oncol. (2021) 11:710899. doi: 10.3389/fonc.2021.710899 34277455 PMC8281889

[106] Chen X Guo C Cui W Sun K Wang Z Chen X . CD56 expression is associated with biological behavior of pancreatic neuroendocrine neoplasms. Cancer Manag Res. (2020) 12:4625–31. doi: 10.2147/cmar.s250071 32606955 PMC7306468

[107] He Y Zhao L Tang X Jiang Q Zhao X Cao Y . Prognostic implications of synaptophysin, CD56, thyroid transcription factor-1, and Ki-67 in pulmonary high-grade neuroendocrine carcinomas. Ann Diagn Pathol. (2024) 68:152239. doi: 10.1016/j.anndiagpath.2023.152239 38006863

[108] Depoilly T Leroux R Andrade D Nicolle R Dioguardi Burgio M Marinoni I . Immunophenotypic and molecular characterization of pancreatic neuroendocrine tumors producing serotonin. Mod Pathol. (2022) 35:1713–22. doi: 10.1038/s41379-022-01110-x 35739266

[109] Chen Q Jiang K Bronze MS Li M Houchen CW Zhang Y . Tumor microenvironment and macroenvironment: a new perspective on holistic oncology. Cancer Lett. (2025) 634:218076. doi: 10.1016/j.canlet.2025.218076 41083101

[110] Rodríguez-Remírez M del Puerto-Nevado L Fernández-Aceñero MJ Cruz-Ramos M García-García L Solanes S . Targeting galectin-1 by aflibercept strongly enhances its antitumor effect in neuroendocrine carcinomas. Neuroendocrinology. (2021) 111:146–57. doi: 10.1159/000506163 31991407

[111] Beyens M Vandamme T Peeters M Van Camp G Op de Beeck K . Resistance to targeted treatment of gastroenteropancreatic neuroendocrine tumors. Endocr Relat Cancer. (2019) 26:R109–30. doi: 10.1530/erc-18-0420 32022503

[112] Dromain C Pavel M Ronot M Schaefer N Mandair D Gueguen D . Response heterogeneity as a new biomarker of treatment response in patients with neuroendocrine tumors. Future Oncol. (2023) 19:2171–83. doi: 10.2217/fon-2022-1137 37497626

[113] Alaklabi S Maguire O Pattnaik H Zhang Y Chow J Wang J . Immune cell molecular pharmacodynamics of lanreotide in relation to treatment response in patients with gastroenteropancreatic neuroendocrine tumors. Cancers (Basel). (2024) 16:3104. doi: 10.3390/cancers16173104 39272962 PMC11394651

[114] Tuncer O Steinberger D Steiner J Hinojos M Rhee SY Humphrey B . Quantitative SPECT/CT metrics in early prediction of [177Lu]Lu-DOTATATE treatment response in gastroenteropancreatic neuroendocrine tumor patients. J Nucl Med. (2024) 65:1584–90. doi: 10.2967/jnumed.124.267964 39266296

[115] Singh S Halperin D Myrehaug S Herrmann K Pavel M Kunz PL . [177Lu]Lu-DOTA-TATE plus long-acting octreotide versus high-dose long-acting octreotide for the treatment of newly diagnosed, advanced grade 2-3, well-differentiated gastroenteropancreatic neuroendocrine tumours (NETTER-2): an open-label, randomised, phase 3 study. Lancet. (2024) 403:2807–17. doi: 10.1016/s0140-6736(24)00701-3 38851203

[116] Raj N Coffman K Le T Do RKG Rafailov J Choi Y . Treatment response and clinical outcomes of well-differentiated high-grade neuroendocrine tumors to lutetium-177-DOTATATE. Neuroendocrinology. (2022) 112:1177–86. doi: 10.1159/000525216 35609558 PMC9671816

[117] Liverani C Spadazzi C Ibrahim T Pieri F Foca F Calabrese C . HRAS overexpression predicts response to lenvatinib treatment in gastroenteropancreatic neuroendocrine tumors. Front Endocrinol (Lausanne). (2023) 13:1045038. doi: 10.3389/fendo.2022.1045038 36743926 PMC9895371

[118] Treppiedi D Catalano R Mangili F Mantovani G Peverelli E . Role of filamin A in the pathogenesis of neuroendocrine tumors and adrenal cancer. Endocr Oncol. (2022) 2:R143–52. doi: 10.1530/eo-22-0055 37435454 PMC10259351

[119] Guo Q Yang W Robinson G Chaludiya K Abdulkadir AN Ghosh Roy F . Unlocking the radiosensitizing potential of MYC inhibition in neuroendocrine Malignancies. Int J Radiat Oncol Biol Phys. (2025) 122:1310–26. doi: 10.1016/j.ijrobp.2025.04.034 40354951

[120] Gillette AA Babiarz CP VanDommelen AR Pasch CA Clipson L Matkowskyj KA . Autofluorescence imaging of treatment response in neuroendocrine tumor organoids. Cancers (Basel). (2021) 13:1873. doi: 10.3390/cancers13081873 33919802 PMC8070804

[121] Zhao J Bera K Mohamed A Li Q Ramaiya N Tirumani SH . Comparison of RECIST 1.1, mRECIST and PERCIST for assessment of peptide receptor radionuclide therapy treatment response in metastatic neuroendocrine tumors. Curr Probl Diagn Radiol. (2025) 54:228–32. doi: 10.1067/j.cpradiol.2024.10.003 39389807

[122] Cao M Huang P Xu L-S Zhang Y-H . Analysis of gut microbiota-derived metabolites regulating pituitary neuroendocrine tumors through network pharmacology. Front Pharmacol. (2024) 15:1403864. doi: 10.3389/fphar.2024.1403864 39295931 PMC11408289

[123] Zheng S-Y Su Y-Y Cai F-L Xu D-F Xu Y-Q . From mechanisms to precision medicine: the role of organoids in studying the gut microbiota-tumor microenvironment axis. Front Microbiol. (2025) 16:1669482. doi: 10.3389/fmicb.2025.1669482 41158775 PMC12555020

[124] Vitale G Dicitore A Barrea L Sbardella E Razzore P Campione S . From microbiota toward gastro-enteropancreatic neuroendocrine neoplasms: are we on the highway to hell? Rev Endocr Metab Disord. (2021) 22:511–25. doi: 10.1007/s11154-020-09589-y 32935263 PMC8346435

[125] Doornebal EJ Harris N Riva A Jagatia R Pizanias M Prachalias A . Human immunocompetent model of neuroendocrine liver metastases recapitulates patient-specific tumour microenvironment. Front Endocrinol (Lausanne). (2022) 13:909180. doi: 10.3389/fendo.2022.909180 35909511 PMC9326114

[126] Argelaguet R Velten B Arnol D Dietrich S Zenz T Marioni JC . Multi-Omics Factor Analysis-a framework for unsupervised integration of multi-omics data sets. Mol Syst Biol. (2018) 14:e8124. doi: 10.15252/msb.20178124 29925568 PMC6010767

[127] Argelaguet R Arnol D Bredikhin D Deloro Y Velten B Marioni JC . MOFA+: a statistical framework for comprehensive integration of multi-modal single-cell data. Genome Biol. (2020) 21:111. doi: 10.1186/s13059-020-02015-1 32393329 PMC7212577

[128] Wang B Mezlini AM Demir F Fiume M Tu Z Brudno M . Similarity network fusion for aggregating data types on a genomic scale. Nat Methods. (2014) 11:333–7. doi: 10.1038/nmeth.2810 24464287

[129] Singh A Shannon CP Gautier B Rohart F Vacher M Tebbutt SJ . DIABLO: an integrative approach for identifying key molecular drivers from multi-omics assays. Bioinformatics. (2019) 35:3055–62. doi: 10.1093/bioinformatics/bty1054 30657866 PMC6735831

[130] Shen R Olshen AB Ladanyi M . Integrative clustering of multiple genomic data types using a joint latent variable model with application to breast and lung cancer subtype analysis. Bioinformatics. (2009) 25:2906–12. doi: 10.1093/bioinformatics/btp543 19759197 PMC2800366

[131] Picard M Scott-Boyer MP Bodein A Perin O Droit A . Integration strategies of multi-omics data for machine learning analysis. Comput Struct Biotechnol J. (2021) 19:3735–46. doi: 10.1016/j.csbj.2021.06.030 34285775 PMC8258788

[132] Hamamoto R Komatsu M Takasawa K Asada K Kaneko S . Epigenetics analysis and integrated analysis of multiomics data, including epigenetic data, using artificial intelligence in the era of precision medicine. Biomolecules. (2020) 10:62. doi: 10.3390/biom10010062 31905969 PMC7023005

[133] Zhang H Chen Y Li F . Predicting anticancer drug response with deep learning constrained by signaling pathways. Front Bioinform. (2021) 1:639349. doi: 10.3389/fbinf.2021.639349 36303766 PMC9581064

[134] Li W Chen C Zhu X Zhang C . Single-cell and spatial multiomics: applications for diseases. MedComm (2020). (2025) 6:e70553. doi: 10.1002/mco2.70553 41403916 PMC12703058

[135] Huan C Li J Li Y Zhao S Yang Q Zhang Z . Spatially resolved multiomics: data analysis from monoomics to multiomics. BME Front. (2025) 6:84. doi: 10.34133/bmef.0084 39810754 PMC11725630

[136] Boons G Vandamme T Peeters M Beyens M Driessen A Janssens K . Cell-free DNA from metastatic pancreatic neuroendocrine tumor patients contains tumor-specific mutations and copy number variations. Front Oncol. (2018) 8:467. doi: 10.3389/fonc.2018.00467 30443491 PMC6221938

[137] Modlin IM Kidd M Falconi M Filosso PL Frilling A Malczewska A . A multigenomic liquid biopsy biomarker for neuroendocrine tumor disease outperforms chromogranin A and has surgical and clinical utility. Ann Oncol. (2021) 32:1425–33. doi: 10.1016/j.annonc.2021.08.1746 34390828 PMC9393904

[138] Al-Toubah T Cives M Valone T Blue K Strosberg JR . Sensitivity and specificity of the NETest: a validation study. Neuroendocrinology. (2021) 111:580–5. doi: 10.1159/000509866 32615553

[139] McShane LM Altman DG Sauerbrei W Gion M Clark GM . REporting recommendations for tumour MARKer prognostic studies (REMARK). Br J Cancer. (2005) 93:387–91. doi: 10.1038/sj.bjc.6602678 16106245 PMC2361579

[140] Collins GS Moons KGM Dhiman P Riley RD Beam AL Van Calster B . TRIPOD+AI statement: updated guidance for reporting clinical prediction models that use regression or machine learning methods. BMJ. (2024) 385:e078378. doi: 10.1136/bmj-2023-078378 38626948 PMC11019967

[141] Pugazenthi S Pari SS Zhang Z Silverstein J Kim AH Patel B . The evolution and application of multi-omic analysis for pituitary neuroendocrine tumors. Front Med (Lausanne). (2025) 12:1629621. doi: 10.3389/fmed.2025.1629621 40959438 PMC12434087

